# Evaluation of an integrated clinical workflow for targeted next-generation sequencing of low-quality tumor DNA using a 51-gene enrichment panel

**DOI:** 10.1186/s12920-014-0062-0

**Published:** 2014-11-14

**Authors:** Ashish Choudhary, Elizabeth Mambo, Tiffany Sanford, Michael Boedigheimer, Brian Twomey, Joseph Califano, Andrew Hadd, Kelly S Oliner, Sylvie Beaudenon, Gary J Latham, Alex T Adai

**Affiliations:** Asuragen, Inc., 2150 Woodward St, Suite 100, Austin, TX 78744-1038 USA; Department of Otolaryngology-Head and Neck Surgery, Milton J. Dance Head and Neck Center, Greater Baltimore Medical Center, Baltimore, MD 21204 USA; Amgen, Inc., 1 Amgen Center Dr, Thousand Oaks, CA 91320 USA; Current Address: Roche Sequencing Unit, 4300 Hacienda Dr, Pleasanton, CA 94588 USA

## Abstract

**Background:**

Improvements in both performance and cost for next-generation sequencing (NGS) have spurred its rapid adoption for clinical applications. We designed and optimized a pan-cancer target-enrichment panel for 51 well-established oncogenes and tumor suppressors, in conjunction with a bioinformatic pipeline informed by in-process controls and pre- and post-analytical quality control measures.

**Methods:**

The evaluation of this workflow consisted of sequencing mixtures of intact DNA to establish analytical sensitivity and precision, utilization of heuristics to identify systematic artifacts, titration studies of intact and FFPE samples for input optimization, and incorporation of orthogonal sequencing strategies to increase both positive predictive value and variant detection. We also used 128 FFPE samples to assess clinical accuracy and incorporated the previously described quantitative functional index (QFI) for sample qualification as part of detailing complete system performance.

**Results:**

We observed a concordance correlation coefficient of 0.99 between the observed versus expected percent variant at 250 ng input across 4 independent sequencing runs. A subset of the systematic variants were confirmed to be barely detectable on an independent sequencing platform (Wilcox signed-rank test p-value <10^−16^), and the incorporation of orthogonal sequencing strategies increased the harmonic mean of sensitivity and positive predictive value of mutation detection by 41%. In one cohort of FFPE tumor samples, coverage and inter-platform concordance were positively correlated with the QFI, emphasizing the need for pre-analytical sample quality control to reduce the risk of false positives and negatives. In a separate cohort of FFPE samples, the 51-gene panel achieved 78% sensitivity (95% CI = 56.3, 92.5) with 100% PPV (95% CI = 81.5, 100.0) based on known mutations at 7.9% median abundance. By sequencing specimens using an orthogonal NGS technology, sensitivity was improved to 87.0% (95% CI = 66.4,97.2) while maintaining PPV.

**Conclusions:**

The results highlight the value of process integration in a comprehensive targeted NGS system, enabling both discovery and diagnostic applications, particularly when sequencing low-quality cancer specimens.

**Electronic supplementary material:**

The online version of this article (doi:10.1186/s12920-014-0062-0) contains supplementary material, which is available to authorized users.

## Background

NGS has been instrumental in accelerating discovery in cancer genomics via whole genome sequencing (WGS), whole exome sequencing (WES), and high-depth targeted amplicon sequencing (TAS). Researchers have used NGS to help identify somatic mutations, understand clonal evolution, and, most recently, advance personalized medicine [1]. Studies of the genomic landscapes of common cancer have revealed that ~95% of non-synonymous mutations are single base variants, rather than insertions or deletions that affect one or several bases [2]. As such, the detection of these single nucleotide variants (SNV) is a primary objective of cancer-related sequencing studies. Large multi-sample studies have resulted in breakthrough discoveries of somatic mutations in the context of clonal evolution, pathway analysis, and associations with gene expression patterns [3,4]. In cancer research, specificity for somatic mutation detection is typically achieved by comparing tumor DNA sequence with a matching normal tissue DNA sequence to differentiate germline and somatic genetic variants [5]. This strategy, however, is not feasible for routine clinical testing. Although the utility of NGS in cancer genomics is rapidly progressing, TAS in formalin-fixed, paraffin embedded (FFPE) specimens has received much less attention in the literature. Preservation of tissue in the form of FFPE is a cost effective traditional method that is widely used for archiving tissue specimens, and provides a large source of archival materials for cancer research.

For oncology diagnostics, TAS offers a number of benefits compared to WGS or WES, including ultra-deep (>1000X) coverage in gene regions linked to professional clinical practice guidelines and targeted therapeutics in clinical trials, increased sequencing depth and analytical sensitivity for variant detection from tumor subclones, improved sample throughput, reduced per sample costs, faster turnaround time, and a lower probability of incidental findings and variants of unknown significance [6]. Unlike WGS or WES, TAS strategies (such as the one described here) can be devised specifically to accommodate FFPE DNA modifications and fragmentation, and thus this approach offers low-abundance mutation detection to address tumor heterogeneity [7–15], even from low-quality FFPE DNA. As a result, TAS has increasingly become the method of choice in clinical laboratories.

Research to date has underlined the potential utility of NGS for individualized and targeted cancer therapy. The past couple of years have seen a rising tide of studies investigating TAS applications on FFPE samples. At first, preliminary studies were limited to 20 or fewer FFPE samples [9,10,12,13]. As the technology burgeoned, applications appeared with more samples [7,16], as well as broader panels based on hybrid-capture technology [8,17], however the depth of analytical characterization has been limited including specialized workflows to specific sequencing technologies [11]. Additional panels for targeted NGS are the subject of growing clinical interest, including recent reports describing the clinical laboratory validation of capture hybridization panels that enrich FFPE DNA [15,18] and a focused thyroid cancer TAS panel [19], as well as a clinical research study using a limited-content TAS panel aimed at very high sensitivity, variant identification in plasma and FFPE specimens [14]. The existing research, however, includes few examples of accurate, sensitive and reliable mutation detection across a well-powered set of FFPE clinical DNA samples, and none that make use of a comprehensive PCR-based enrichment workflow with associated controls, QC, and bioinformatic tools.

The goal of this study was to provide an analytical framework to complement the development, evaluation, and application of a comprehensive system for targeted NGS. This system was specifically designed to enable the identification of clinically actionable mutations in FFPE DNA known to be relevant across many different cancer pathologies. To achieve this objective, we first designed mixtures of well-characterized cell line DNA enriched for low-level variants, yet covering a range of allele frequencies sufficient for establishing assay linearity and accuracy. A unique method for identifying systematic variants (SVs) – that is, artifacts not of biological origin – was developed and used to filter results. The platform was then used to analyze residual clinical FFPE specimens in order to refine variant detection strategies, particularly in samples with a high background of FFPE DNA “noise,” such as false positives presenting as G > A or C > T transitions (to be concise, we will use GC > AT to represent G > A or C > T transitions). The large-scale target enrichment and data analysis platform was then evaluated with respect to: 1) sample DNA qualification; 2) platform accuracy determination, with and without confirmation testing; and 3) quantification of the upper limits of analytical validity. The insights gleaned from the development and validation of this system extend the knowledge base of TAS in FFPE DNA, and broaden the foundation for novel biological discoveries and diagnostic detection using this and similar methods. Finally, our approach extends beyond the optimization of discrete steps in the TAS workflow, such as the enrichment chemistry or back-end bioinformatics, to implement and integrate controls and in-process quality measures across pre-analytical, analytical and post-analytical test phases. Although we did not address such critical topics such as small indel detection, we demonstrate that this holistic approach improves the accuracy of variant detection, quantification, and interpretation, and addresses the need for standardized and validated methods for the routine use of TAS in clinical oncology.

## Methods

### Clinical specimens

Lymphocytes and tumor specimens were collected from head and neck squamous cell carcinoma (HNSCC) patients at Johns Hopkins Medical Institutions. Tissue was collected after patients were enrolled in a Johns Hopkins Institutional Review Board Protocol, and informed consent was obtained from all patients prior to enrollment and collection of tissues. Appropriate informed consent was obtained after institutional review board approval. Prior to use, all specimens were stored in liquid nitrogen. Lymphocytes were digested using standard SDS/proteinase K protocols and resulting DNA was purified using standard phenol-chloroform extraction and ethanol precipitation. DNA was resuspended in LoTE buffer (EDTA 2.5 mM and Tris–HCl 10 mM, pH 7.5), and DNA concentration was quantified using the NanoDrop ND-1000 spectrophotometer (Thermo Scientific). The resulting lymphocyte DNA was sent to Asuragen for targeted NGS analysis.

The 46 FFPE colorectal cancer DNA sample set was comprised of DNA extracted from 26 individual tumor specimens using the QIAamp DNA FFPE Tissue Kit (Qiagen, Valencia, CA, USA). These 26 DNA samples were used to create 24 DNA mixtures (KRAS codon 12/13 mutant diluted with KRAS codon 12/13 wild-type) and 22 neat samples. The 46 colorectal samples, as well as the underlying 26 individual samples, had all been previously screened for KRAS codon 12/13 status through various, repeated methods, including Sanger sequencing and the allele-specific PCR assay (DxS/Qiagen; Manchester, UK) (data not shown). The 26 tumor specimens were all colon or rectal adenocarcinomas obtained from Asterand (Detroit, MI, USA).

The remaining colon cancer FFPE tissue specimens come from stage II tumors that were acquired from FolioBio (Columbus, OH). All specimens were residual de-identified samples that were procured in accordance with appropriate human subjects’ regulations using a protocol that was approved by an institutional review board. Moreover, Asuragen has filed a Federalwide Assurance for the Protection of Human Subjects (FWA) with the US Department of Health and Human Services. Prior to nucleic acid isolation, a hemotoxylin and eosin (H&E)-stained slide representing the FFPE tissue block was prepared and reviewed by a board-certified anatomic pathologist at Asuragen to assess specimen quality and identify areas with a high proportion of cancer cells. Specimens were macro-dissected to achieve at least 80% tumor content. DNA was isolated from the enriched FFPE sections using the RecoverAll Total Nucleic Acid Isolation Kit for FFPE (Life Technologies) according to the manufacturer’s instructions. DNA was quantified using the Nanodrop 1000 (Thermo Scientific).

A total of 72 FFPE thyroid samples were purchased from Asterand (Asterand Plc., Detroit, MI, USA). The benign samples included 9 follicular adenoma, 5 oncocytic FA, 10 hyperplastic nodules, 4 multinodular goiter, and 2 cases of Hashimoto’s disease. The malignant samples included 18 papillary thyroid carcinoma of classical type, 3 oncocytic PTC, 1 columnar PTC, 8 follicular variant of PTC, 10 follicular thyroid carcinoma, 1 oncocytic FTC, and 1 medullary thyroid cancer. For each sample, H&E slides were prepared and reviewed by a pathologist at Asuragen to confirm the histological diagnosis and establish tumor content. All samples with less than 50% tumor were marked for tumor enrichment by macrodissection. DNA and total RNA were isolated using the RecoverAll Total Nucleic Acid Isolation Kit for FFPE (Life Technologies). DNA from each sample was interrogated for the presence of distinct genetic alterations included in the miRInform™ Thyroid panel (Asuragen, Austin, TX); however, we excluded translocations from the analysis.

### Design of the 1052-amplicon panel

We designed and evaluated a 1052-target enrichment panel to amplify 109,302 genomic positions across 51 genes for use in NGS analysis (Table 1). PCR primer sets in the panel were designed for targeted sequencing in 28 genes and exome sequencing of the remaining 23 genes. The genomic coordinates of coding exons were submitted to RainDance Technologies for design and synthesis of target-specific PCR primers for amplification of 70–200 bp products. We initially evaluated the panel by sequencing 16 fresh-frozen samples to identify regions with poor sequencing coverage (amplicons that yielded 100 or fewer reads across all 16 samples). Alternative PCR primer sets were designed to improve sequencing in these poor sequencing-coverage regions. The final TAS panel included a total of 1052 amplicons.

**Table 1:** **Codon and gene coverage of the 1052-amplicon panel**

**Gene**	**Transcript**	**Codon**
ABL1	ENST00000318560	85–505
AKT1	ENST00000349310	1–96, 146–189, 277–391, 455–481
AKT2	ENST00000392038	192–320
BRAF	ENST00000288602	393–664
CDH1*	ENST00000261769	1–883
CDK4	ENST00000257904	1–73, 228–273
CDKN2A*	ENST00000446177	1–153
CEBPA*	ENST00000498907	1–359
CREBBP	ENST00000262367	1–29, 853–929, 1328–1465, 1725–1761, 2196–2299
CTNNB1*	ENST00000396185	1–782
EGFR*	ENST00000275493	1–1211
ERBB2	ENST00000269571	147–215
FES	ENST00000328850	72–223
FGFR1*	ENST00000447712	1–823
FGFR3	ENST00000340107	206–760
FLT3	ENST00000241453	437–685, 807–885
FOXL2	ENST00000330315	91–158
GATA1	ENST00000376670	1–200
GNA11	ENST00000078429	202–245
GNAQ	ENST00000286548	159–245
HIF1A*	ENST00000337138	1–827
HRAS*	ENST00000397594	1–150
IDH1	ENST00000345146	41–174, 284–331
IDH2	ENST00000330062	125–178
IKBKB	ENST00000520810	311–375, 580–662
JAK2	ENST00000381652	443–711, 858–903
KIT*	ENST00000288135	1–977
KRAS*	ENST00000256078	1–150
MEN1*	ENST00000377326	1–611
MET	ENST00000318493	981–1330
MPL	ENST00000372470	180–230, 440–522
NF2*	ENST00000361166	1–579
NOTCH1*	ENST00000277541	21–2556
NPM1	ENST00000393820	47–118
NRAS	ENST00000369535	1–97
PAX5	ENST00000358127	71–137, 304–367
PDGFRA	ENST00000257290	552–960, 1041–1090
PIK3CA	ENST00000263967	21–106, 301–353, 418–582, 672–729, 889–1069
PIK3R1*	ENST00000521657	1–725
PTCH1*	ENST00000331920	1–1448
PTEN*	ENST00000371953	1–404
PTPN11	ENST00000351677	46–111, 483–533
RB1*	ENST00000267163	1–491, 500–929
RET	ENST00000355710	627–694, 870–934
SMAD4*	ENST00000342988	1–302, 319–553
SMARCB1*	ENST00000263121	1–386
SMO*	ENST00000249373	1–788
SRC*	ENST00000445403	1–537
STK11*	ENST00000326873	1–434
TP53*	ENST00000269305	1–394
VHL	ENST00000256474	1–52, 104–214

The table shows the list of genes, transcripts and codons targeted by the 1052-amplicon panel. In sum, the panel covers over 10,000 mutations annotated in v64 of the COSMIC database, including single nucleotide variants (SNVs) and small indels up to 2 bp. The transcript models used for codon identification are based on Ensembl Genomes release 17 (GRCh37). *All codons are covered in the genes marked with an asterisk.

### DNA sample preparation and sequencing

Intact genomic DNA from cell lines and frozen tissues was sheared to an average size of ~4 kb using the Covaris S220 focused-ultrasonicator (Covaris, Woburn, MA). The sheared DNA and DNA from FFPE specimens were quantified using the Nanodrop 1000 (Thermo Scientific, DE). A fraction of the DNA (100 ng) was used to evaluate fragmented DNA size ranges using the E-gel system (Life Technologies). Genomic DNA (250 ng, 500 ng or 2,000 ng) was merged with the 1052-amplicon panel using the RDT 1000 instrument (RainDance Technologies, MA). The merged droplets were amplified by PCR using the following conditions: denaturation at 94°C for 2 min; 55 cycles of 94°C for 15 s, 54°C for 15 s, 68°C for 30 sec; final extension at 68°C for 10 min, and 4°C hold. After breaking the emulsion, the resulting PCR products were purified using the MinElute kit (Qiagen) according to the manufacturer’s instructions. A fraction of the target-enriched DNA was then evaluated for size and quantity using a Bioanalyzer Lab-on-a-chip DNA 12000 (Agilent Technologies) and Nanodrop spectrophotometer (Thermo Scientific), respectively. Next, a tagging PCR reaction was performed to append unique barcode sequences to each sample and to add adapters needed for sequencing on the GAIIx/HiSeq platforms (Illumina). Barcodes were chosen from a set of 48 standard barcode sequences obtained from Illumina. Purified target-enriched DNA (10 ng) from the initial PCR was amplified in the tagging PCR with the following thermal cycling conditions: 94°C for 2 min, 10 cycles of 94°C for 30 s, 56°C for 30 s, 68°C for 1 min; 68°C for 10 min, and 4°C hold. Tagged PCR products were pooled and purified using the MinElute PCR purification kit (Qiagen) according to the manufacturer’s instructions. The products were quantified using the KAPA Library Quant kit (KAPA Biosystems, South Africa) as per manufacturer’s instructions. All samples were normalized to 8.6 nM, and pools of 8 to 15 samples per lane for the GAIIx and 32 samples per lane for the HiSeq were prepared. Flow cell preparation and data acquisition were completed using Illumina’s recommended protocols. Paired-end sequencing runs (2x151) were performed using the Illumina GAIIx and HiSeq platforms.

### AmpliSeq cancer panel and PGM sequencing

For comparative studies, a subset of our samples was also enriched for specific target sequences using the Ion AmpliSeq Cancer Panel 1.0 (Life Technologies) according to the manufacturer’s protocol. Briefly, 10 ng of cell line or FFPE DNA samples were pre-amplified in 19 cycles of PCR (98°C for 15 s and 60°C for 4 min) and the products were purified using magnetic bead purification (Agencourt AMPure XP, Beckman Coulter). The purified PCR fragments were 5’ phosphorylated and ligated to the adaptor needed for emulsion PCR using the Ion Torrent system (Life Technologies). The AMPure-purified ligation products were nick translated at 72°C for 1 min and amplified using 10 cycles of PCR (98°C for 15 s, 60°C for 4 min). The amplified library products were purified using magnetic bead purification (Agencourt AMPure XP, Beckman Coulter) and quantified using a Bioanalyzer and High Sensitivity DNA chip (Agilent Technologies). Approximately 44 million copies of library DNA were used for emulsion PCR on the Ion One Touch Instrument (Life Technologies), and 50% of enriched products were loaded onto Ion 316 chips for sequencing on the Ion PGM system (Life Technologies).

### Reference DNA mixtures for evaluating platform linearity and accuracy

**Figure 1:**
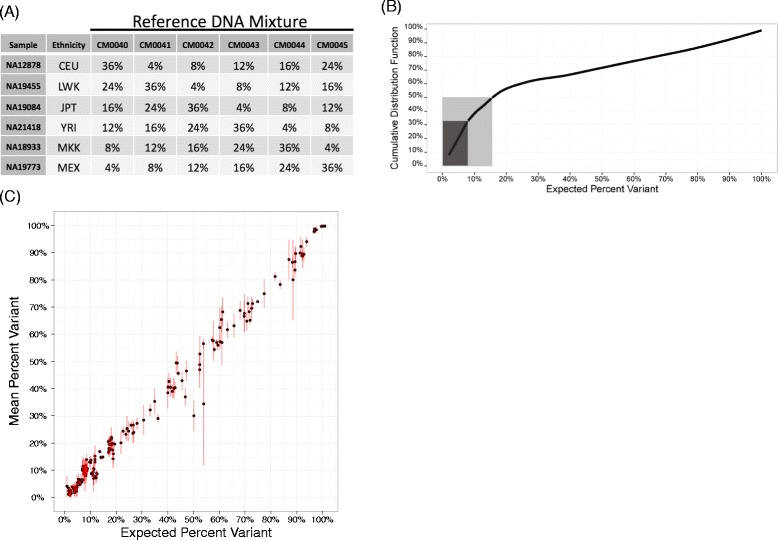
**Reference DNA mixtures are an analytical resource for evaluating platform linearity and accuracy. A)** The table shows the mixing coefficients of each cell line DNA sample (rows) used to create the specified reference DNA mixtures (columns). An optimization procedure was used to select the most diverse samples based on genetic-variant annotation across the 1052-amplicon panel, and the mixing coefficients were set to enrich for variants at <20 percent variant. **B)** The cumulative distribution function of the percentage of variants (y-axis) captured by the reference DNA mixtures as a function of the expected percent variant (x-axis). The dark box shows that 1/3 of the variants in the mixtures are expected to be present at ≤8% and the lighter box shows that 50% of the variants are expected to be present at ≤16% variant. **C)** The mean percent variant (y-axis) as a function of the expected percent variant (x-axis) for the 195 expected variant sites across 4 separate sequencing runs using 250 ng DNA input. Each point is the mean and a red vertical line spans the range of the mean ± standard deviation. A small jitter (less than 1%) was added to the expected percent variant (x-axis) in order to minimize over plotting.

We designed a set of reference DNA mixtures from cell lines with known genetic variants that could be used to quantitate platform precision, linearity and accuracy. Using the genotypes made available by the 1000 Genomes Project [20], we selected a set of diverse cell lines from the group of 1192 samples available at ATCC/Coriell as follows. The search was seeded with the NA12878 cell line, and samples were added one-by-one to maximize loci diversity across the genomic regions queried by our 1052-amplicon library. A locus was considered diverse if the genotype was not represented in the set of samples already selected. This procedure inherently selected samples from diverse ethnicities: NA12878, NA18933, NA19084, NA19455, NA19773, and NA21418. The selection process relied on annotations from the 1000 Genomes Project, but the variant annotations were incomplete; thus, all 6 samples were individually sequenced at high depth to unambiguously identify variants and establish true genotypes (>3000 reads, with 97% of the bases within 5 fold of median sequencing depth). Figure 1A shows the mixing coefficients and order of samples used to provide good statistical power for evaluation of low-level variant detection (Figure 1B). The sequencing data for these samples is available from the Sequence Read Archive (SRA) as Bioproject PRJNA257348. Although we didn’t perform indel analysis in this study, a similar process can be used for evaluating indel callers, however, more mixtures will likely be necessary to derive a comparable number of abberations.

### Sample qualification using the quantitative functional index

In general, QFI estimations were performed as previously described [21]. DNA extracted from FFPE samples is often degraded and further compromised with chemical modifications such as cross-linking, deamination and adducts. A novel, real-time PCR based assay, referred to as quantitative functional index (QFI) was used to assess the proportion of amplifiable templates in these degraded samples [21]. DNA samples were quantified using a NanoDrop spectrophotometer (Thermo Scientific) and normalized to 5 ng/μL in deionized water. Samples were then assessed using qPCR on a 7900HT Fast Real-Time PCR System (Life Technologies). Quantification of amplifiable DNA was assessed by amplifying a 180 bp region in the ferritin, heavy polypeptide 1 (*FTH1*) gene. The assay was chosen for two reasons; 1) the amplicon length of the interrogating gene, *FTH1* (180 bp), was similar to the median length (171 bp) of the library generated using the 1052-amplicon panel, and, 2) the *FTH1* gene lacks widespread gene amplifications or deletions across a spectrum of common and uncommon cancers [21]. For example, an analysis of copy number changes through the cBioPortal for Cancer Genomics revealed an overall deletion/amplification rate of <1% for all sample types tested for the *FTH1* gene [22,23]. qPCR was carried out in 11 μL reactions with 1X TaqMan Gene Expression Master Mix (Life Technologies), 1X *FTH1* primer/probe mix (Hs01694011_s1; Life Technologies) and 5 ng gDNA. The PCR cycling conditions were 95°C for 10 min, 50 cycles of 95°C for 15 s and 60°C for 1 min. A high-quality genomic DNA, NA04025 (Coriell Cell Repositories, Camden, NJ, USA), was used to generate a calibration curve using a 5-fold titration series, from 50 ng to 16 pg. PCR-competent copy number was then calculated from the calibration curve and reported as QFI.

### Bioinformatics

#### General workflow

The raw sequence read data generated from the GAIIx were demultiplexed and preprocessed using Illumina’s CASAVA software package (1.7) to produce sample-specific FASTQ files. Additional processing was similar to previously published studies [24]. Briefly, the sequencing adaptors and target-specific primers were trimmed. We further trimmed the reads to retain only high-quality data (Q20 or higher). Paired-end alignments were performed using the BWA aligner (0.5.9-r16) [25] against the human genome (hg19). In the case of data generated from the PGM, TMAP (tmap.2.X) (https://github.com/iontorrent/TMAP) was used as to align sequences to the human genome. In either case, alignments were post-processed using a GATK (1.3-21)-based workflow to add read-group information, perform local realignments, recalibrate Q scores, and estimate base alignment scores (BAQ scores) as described previously [24,26,27]. Note that default GATK genotyper parameters were used except PCR duplicates were not removed and subsampling was turned off (in the context of TAS all reads corresponding to an amplicon will have the same start and stop position). Other bioinformatics analyses, including database annotation and variant calling, were based on previously published research [24]. An overview of the analysis workflow is captured in Additional file 1: Figure S7. All analyses were performed using the R programming language.

#### Identification of systematic variants

Library preparation and sequencing on the Illumina platform are known to introduce systematic variants (SVs) [28] that tend to inflate the false positive rate (FPR) of predicted variants [10,17]. To identify SVs in sequence data generated with TAS using the 1052-amplicon panel, DNA from a set of 29 disease-free lymphocyte samples was sequenced. In this sample set, the annotated SNPs tended to have a standard deviation as a function of the mean that followed Hardy-Weinberg equilibrium. As a result, variants that violated Hardy-Weinberg equilibrium were categorized as SVs. For a given *non-reference* allele (genetic variant) with penetrance *p*, that adheres to the Hardy-Weinberg principle, the relative genotype frequencies within the sample population are predicted to be wild type, heterozygous variant, and homozygous variant *(1-p)*^*2*^, *2p(1-p)* and *p*^*2*^, respectively. The corresponding standard deviation (SD) of the variant penetrance is $SD(p)=\sqrt{p\left(1-p\right)/2}$. Simulations (n = 100,000) were performed by creating samples from a multinomial distribution over a range of penetrance estimates to produce the expected number of observations of each genotype. A sampling was then made of the percent variant estimates corresponding to the appropriate genotypes in order to estimate the mean and SD of the penetrance. Variants with the following criteria were defined as SVs: no annotation in dbSNP or COSMIC, exceeded 3 SD of variant penetrance, present with a minimum of 2% variant in at least 1 sample.

#### Adaptive thresholds for variant calling

The most convenient variable we modulated to balance sensitivity and PPV was the variant score threshold, which could be decreased to improve sensitivity or increased to improve PPV. Once a threshold is set, positions in the panel with scores above the threshold are deemed predicted variants, whereas those below the threshold are not. However, FFPE samples require separate thresholds for GC > AT transitions and all other substituions so thresholds were determined in a two-dimensional space or grid. In this study, a threshold setting strategy (grid search) based on three constraints was established:

1. Calls per kilobase: Variant prediction (both somatic and germline) would occur at 0.36 to 1.5 variants per kb, a rate dominated by germline variation (~1 variant per kilobase [29–31]) as opposed to somatic mutations (~1–10 mutations per megabase [4]).
2. Percent annotated: At least 75% of variants would be annotated by publicly available resources [20] such as dbSNP [32] and COSMIC [33,34]. We assumed that the percentage of annotated variants to be a surrogate for positive predictive value (PPV).
3. Ti/Tv: We would expect variants to occur with a ratio of transitions to transversions (Ti/Tv) in the expected range of 1.58 to 4.53 [35].

The three metrics identified above were computed at evenly spaced intervals over a grid of variant score thresholds, and the set of thresholds that satisfy all constraints was identified. In order to select a single threshold pair, we selected the threshold that was closest to the default fresh-frozen threshold of (6,6). Conveniently, this strategy is independent of the methodology used to calculate score variants, and can work for any method producing a continuous value. We know from previous studies [13,36] and the current study, that GC > AT transitions are inflated due to the fixation process. Therefore, the constrained optimization was run as a grid search, with GC > AT thresholds estimated independently of other substitutions.

## Results and discussion

**Figure 2:**
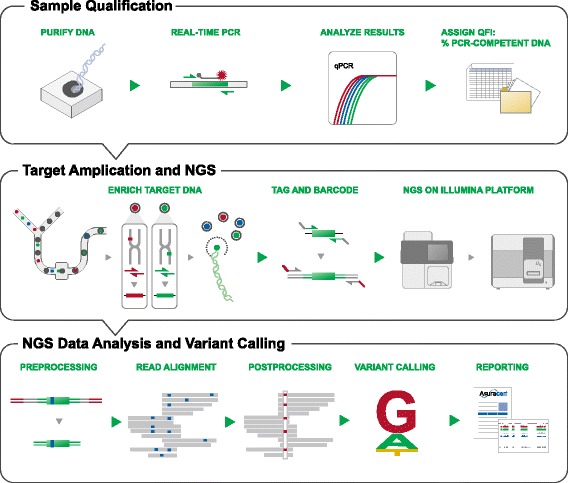
**Technology overview of NGS using the 1052-amplicon targeted sequencing panel.** Sample qualification is performed using a real-time PCR assay to determine the percent “functional” DNA copies in FFPE samples using the Quantitative Functional Index (QFI). The library preparation is based on RainDance technologies picodroplet PCR to amplify target sequences with subsequent sequencing on an Illumina platform (GAIIx or HiSeq). The bioinformatics workflow trims adaptors and filters out low-quality reads, then includes genomic alignments, variant calling and result reporting (See Materials and Methods for more details and Additional file 1: Figure S6 for the result visualization).

In this study, we designed and assessed a targeted cancer TAS panel covering 51 genes (Table 1) and an integrated bioinformatic analysis system for detecting and quantifying mutations. Using calibrated mixtures of cell line and residual clinical FFPE DNA we evaluated and optimized the analytical and clinical performance of the system. Figure 2 shows the sample analysis workflow including: 1) functional DNA sample quantification and quality control using real-time PCR; 2) target enrichment using massively parallel picodroplet PCR; 3) NGS using multiple instruments, including the Illumina GAIIx and HiSeq, and Personal Genome Machine (PGM) for confirmation studies; and 4) bioinformatics processing including read preprocessing, alignment, variant calling and result visualization (see also reference [24]). The TAS enrichment panel is sufficiently broad to enable novel cancer-associated variant discovery while also providing the sequencing depth required to detect low-abundance mutations raising implications for clinical decision-making using targeted therapeutics (see Table 2 for an overview of the samples and objectives for the current study). The TAS panel and integrated workflow were designed and optimized to address all key phases of test performance, including pre-analytical (e.g., use of quantitative functional index PCR (QFI™-PCR, hereafter referred to as QFI) [21] for sample qualification and risk mitigation), analytical (e.g., accuracy, precision, sensitivity and specificity using intact cell line DNA and lower quality DNA from FFPE samples), and post-analytical (e.g., development of robust bioinformatic pipelines and reporting across analytical and clinical samples, including processing of 128 distinct FFPE samples across 3 sample cohorts and encompassing a concordance analysis among multiple mutation-detection technologies such as liquid bead array and qPCR).

**Table 2:** **Overview of study objectives and strategy**

**Phase**	**Primary objectives**	**Sample set**	**# Samples**	**Sample type**	**DNA input (ng)**	**1052-Amplicon sequencing platform**	**Confirmation sequencing platform and panel**
Workflow Optimization And Platform Evaluation	Evaluate analytical performance of the panel	Reference DNA mixtures with known genotypes (based on 1000 Genomes Project)	6 (Samples), 6 (Mixtures)	Intact	250–2000	GAIIx	PGM AmpliSeq
	Determine the impact of DNA input quantity						
	Catalog systematic variants based on Hardy-Weinberg equilibrium.	Intact disease free lymphocyte samples	29	Intact	500	GAIIx	N/A
	Evaluate intact- and FFPE-DNA samples	NA12878 and 2 CRC blocks	1 (Intact), 2 (FFPE)	Intact, FFPE	250–1000	GAIIx	N/A
	Demonstrate the impact of input DNA mass on variance						
	Investigate GC > AT background	CRC blocks	8	FFPE	250	GAIIx	GAIIx 35-amplicon
Platform Evaluation Using Clinical Specimens	Assess performance on clinically relevant FFPE samples	Clinical CRC samples	46	FFPE	250	GAIIx	PGM 1-amplicon
	Assess performance on clinically relevant FFPE samples stratified by QFI	Clinical thyroid-cancer samples	72	FFPE	500, 1000	HiSeq	Luminex liquid bead array

The first phase of this study was focused on the1052-amplicon panel design, analytical performance testing, and bioinformatics workflow optimization. It was conducted using DNA from intact cell lines with known allele frequencies for analytical and variant detection analysis, and FFPE samples for evaluating platform behavior with low DNA quality samples. The second phase focused on clinical application using FFPE samples from different patient cohorts. Note that amplifiable FFPE samples were quantified using QFI. If an alternative (confirmation) platform was used on a given cohort, both platforms are listed in the last column.

### Assessing analytical performance using cell mixtures

To evaluate the baseline analytical performance of the 1052-amplicon panel and tune and assess appropriate data analysis methods, we created 6 reference DNA sample mixtures from high quality cell line DNA with known genetic variant frequencies. Coincident with recent studies [15,37], we used information available from the 1000 Genomes Project to formulate DNA mixtures that produced 195 expected genetic variants for each. Experiments that included all six mixtures were performed to assess the linearity and detection accuracy of 6*195 = 1170 variants. Input amounts were 2 μg (the original specification for the RDT-1000 picodroplet PCR library prep) for the 6 reference DNA mixtures. Two of the 6 DNA mixtures, CM0042 and CM0045, were also tested at 500 ng and 250 ng input.

Figure 1A shows the mixture coefficients for the reference samples and Figure 1B shows the resulting cumulative distribution function of the expected percent variant (e.g., minor allele frequency or SNP fraction). The NGS loading strategy was designed to provide a sequencing depth of ~2000 reads per base on an Illumina Genome Analyzer IIx instrument running 8 samples per lane, with 20–30 million reads generated per lane. The 1052-amplicon panel interrogates 109,302 genomic positions corresponding to 327,906 possible single-nucleotide variant hypotheses (3 possible transitions or transversions for each position). Unless otherwise stated, all subsequent analyses were performed in the context of a hypothesis that represents one of three possible base substitutions at any given position in the panel.

To estimate baseline analytical performance at the amplicon level, we first summarized median amplicon sequencing coverage across the 6 reference mixtures at 2 μg input. At this DNA input, 92% of the amplicons were covered within 5-fold of the median depth (2,663 reads), and 89% were represented within 2-fold of the median. These values are consistent with high coverage uniformity, which is required for any clinically-oriented cancer panel. Interestingly, when considering the amplicons that had a median sequencing depth greater than 100 reads, sequencing depth was more linearly associated with sequence entropy (Spearman rank correlation (SRC) of 0.53) than amplicon length (SRC = −0.29) or %GC (SRC = −0.46) (Additional file 1: Figure S1). Consistent with previously reported research [11], we observed a negative association between sequencing depth and amplicon length. Like others [9,12], we also observed a lower sequencing depth in amplicons with high and low %GC. We surmise that this result is due to low sequence diversity in those amplicons. For the genomic positions enriched using this panel, 76% and 97% were sequenced to depths within 2-fold and 5-fold of the sample median, respectively, with 84% of positions sequenced to a depth ≥2000 reads, which was the targeted sequencing depth.

To understand the gene panel’s performance across a range of potential allele frequencies, we excluded all positions except the 195 expected variants per mixture. We evaluated the panel’s accuracy and linearity with different quantities of input DNA using the concordance correlation coefficient (CCC) between the observed and expected percent variant per sample (Additional file 1: Figure S2). The CCC metric takes into account both accuracy and precision [38,39]. Filtering out positions with <100 reads and <0.1 of the sample median depth of coverage, we found that all CCC values from TAS with DNA input-mass amounts >250 ng exceeded 0.99 (0.99 without filtering), whereas CCC estimates dropped to 0.98 (0.97 without filtering) at 250 ng input DNA. Finally, we also evaluated the stability of linearity and accuracy in the context of run-to-run variability (Figure 1C). One mixture, CM0045, was sequenced with 250 ng DNA input on 4 separate runs (4 different days) and the CCC estimate between mean percent variant and expected percent variant was 0.99 (0.99 without filtering). Nine of the 195 expected variants failed to produce reads across all 4 runs, with 2–5 variant dropouts per run. This lapse only occurred for mixtures sequenced with a DNA input of 250 ng. The depth of coverage filtering removed approximately 0%, 1% and 2% of the 195 known variants for TAS data from runs with 2000 ng, 500 ng and 250 ng input DNA, respectively.

To quantify the precision of low-level variant calls, we examined variants known to be present at 2% based on the cell mixture coefficients (179 measurements spread across 93 expected variants with all mixtures and inputs). When 2000 ng of input DNA was used for the TAS (all 6 mixtures), the median of the median-estimates for percent variants was 1.99%, while results of 2.31% and 2.39% were seen with lower amounts of input DNA, 500 ng (2 mixtures) and 250 ng (2 mixtures), respectively. The median of the interquartile ranges was 0.74%, 1.32% and 1.52% for 2000 ng, 500 ng and 250 ng DNA input, respectively.

### Identifying platform specific systematic variants

**Figure 3:**
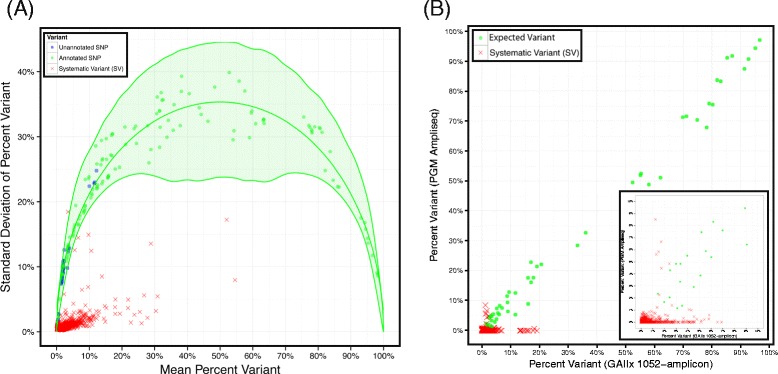
**Identification of systematic variants. A)** The graph shows the behavior of genetic variants following a parabola (green line with a green ribbon for 99.8% CI based on 100,000 simulations) as motivated by Hardy-Weinberg equilibrium. The confidence intervals mostly capture known variants (green circles), but also some unannotated variants (blue circles). In stark contrast, potential variants identified as Systematic Variants (SVs, red Xs) behave differently as the standard deviation of the percent variant as a function of the mean violates Hardy-Weinberg equilibrium. **B)** A subset of SVs were confirmed on an independent sample set (reference DNA mixtures described in Figure 1), sequenced using an alternate NGS platform (Ion PGM, Life Technologies) and TAS panel (AmpliSeq Cancer Panel, Life Technologies). The points show the concordance of the known variants (green circles) on both platforms and SVs identified from 29 intact samples (red X’s). The SVs were predominately plotted along the x-axis (near y-intercept = 0) suggesting they are artifacts specific to the 1052-amplicon panel.

Systematic variants (SVs) – artifacts that are not of biological origin and likely attributed to library preparation or sequencing – are false positives that are observed in multiple samples. To identify SVs, we sequenced intact lymphocyte DNA from 29 subjects with head & neck squamous cell carcinoma. Figure 3A shows a plot of the genotype standard deviation as a function of the mean as modeled by the Hardy-Weinberg equilibrium (HW) [40]. This analysis revealed 274 variants consistent with HW within the 99.8% confidence interval (3 standard deviations), of which 203 (75%) were annotated in dbSNP v132. The number of potential substitutions that fell outside the 99.8% confidence intervals and had a minimum 2% percent variant in at least 1 sample was 2,838. Of these, only 77 (2.7%) were annotated in dbSNP v132 and were therefore excluded from the SV listing. Because the sequencing panel included 109,302 positions (327,906 testable hypotheses), the 2,838-77 = 2,761 SVs comprised just 2.4% of the genomic positions covered by the panel, and 0.8% of the testable hypotheses, but a large potential to have a negative influence on PPV.

To validate the method used to identify SVs, we sequenced a subset of the SVs identified using an orthogonal sequence enrichment method (AmpliSeq® Cancer Panel, Life Technologies) and an independent sample set (the 6 previously defined reference mixtures, which were not used in the identification of the SVs) (Figure 3B). We chose an independent sample set for SV validation to ensure that any assumptions regarding SVs derived on the lymphocyte samples could translate to an entirely different set of samples. This split-test design ensures that bias is minimized and SVs are not specific to a given set of samples. The AmpliSeq panel covers 13,560 bases (excluding primer sequences) of which 8,727 (64%) overlap with our 1052-amplicon panel. Before additional processing, we removed all positions with sequencing depths not within 10-fold of the median from the analysis, so that our SV performance findings were not based on percent variant estimates with insufficient sequencing depth. After filtering, sequencing data were available for 99 unique SVs in the 6 reference mixtures, resulting in 508 measurements. The median percent variant of the 508 SVs using the 1052-amplicon panel was 0.52%, with an interquartile range (IQR) of 1.32%, but a median of 0.00% with an IQR of 0.18% was observed based upon NGS following AmpliSeq enrichment. Overall, we found that 82% of the SVs exhibited a lower percent variant estimate after AmpliSeq enrichment compared to results after enrichment with the 1052-amplicon panel (Wilcox signed-rank test p-value <10^−16^). Of course, these low-level SVs would ordinarily be filtered out by variant calling utilities, but this experiment demonstrated that the relatively higher background for these positions are panel- and library preparation–specific.

SVs were also considered in the larger context of variant calling algorithms. Using the same 6 reference-DNA mixtures, the average precision was calculated for the Poisson caller [24], UnifiedGenotyper [26,27] and VarScan [41], with and without sequencing depth and SV filtering (Additional file 1: Figure S3). According to mixed-effect modeling with SV filtering, variant callers and sequencing-depth filtering as random effects and cell mixtures as fixed effects, 10% of the variation in average precision can be attributed to SV filtering, while 17% is attributed to sequencing-depth filtering and 57% to the variant caller. The remaining 16% variation can be attributed to error from the mixed-effect model. Since variant callers explain the greatest amount of variation in the average precision, mutation detection will greatly benefit from future development in related algorithms. The fact that 17% of the variation can be explained by simple coverage filtering is important as it is a very simple procedure to perform with a significant impact on the results. SV explain 10% of the variation, and this is a substantial yet relatively less important variable for future research development. In aggregate, the relative impact on mutation detection is increasingly SV analysis, coverage filtering and variant prediction algorithms.

Position filtering offers an opportunity to relieve the false positive rate at the risk of reducing sensitivity. Although some studies detail specific criteria for filtering [11], it is usually unclear whether or not all predicted variants in a panel are reported, irrespective of whether annotation exists in publicly available resources such as dbSNP or COSMIC. We repeated the average precision estimates for variant detection by excluding all unannotated sites according to dbSNP or COSMIC (Additional file 1: Figure S3). This filtering procedure excludes over 98% of all possible variants enriched using the 1052-amplicon panel, but it substantially improves the predictive power of all tested callers. Speculatively, the improvement in performance is akin to the power of replication as a site is annotated in public databases because it was previously identified as a variant in the literature. Although excluding unannotated sites can reduce sensitivity particularly for novel variants, it can be justified in the context of clinical reporting where actionable and interpretable results are important for patient management.

### Using independent sequencing technologies to improve sensitivity and positive predictive value

**Figure 4:**
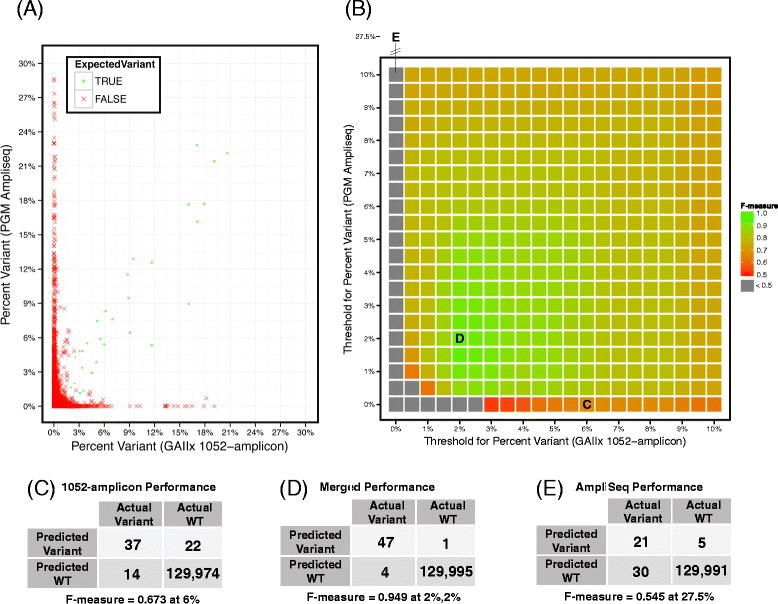
**Independent sequencing of reference DNA mixtures exposes true variants and platform-specific false positives. A)** The percent variant of the 6 reference DNA mixtures as measured from sequencing using the 1052-amplicon (x-axis) and the AmpliSeq (y-axis) panels. The expected variants are green circles and all others are identified with a red X. Variants detected with both workflows fall close to the y = x line, while platform specific false positives (FP) are plotted along the x = 0 axis (AmpliSeq false positives) and y = 0 axis (1052-amplicon panel false positives). **B)** A heatmap of the f-measures (the harmonic mean of sensitivity and PPV) from the two panels for variant calling using different thresholds for percent variant. Each point in the heatmap is colored by the f-measure with green positions having the highest performance. Positions with an f-measure <0.5 are colored gray to prevent color saturation for higher values. The variant calling performance of the 1052-amplicon panel alone is maximized at 6% (label C), but performance can be significantly improved by relaxing threshold constraints for variant calling and considering information from both panels or workflows (label D). In this figure, the systematic variants are not removed from either platform so that the analysis can further illustrate the complementary behavior of each platform. The AmpliSeq panel alone would have performance maximized at position 27.5% (label E). **C)** The 2x2 table showing maximum variant calling performance for samples sequenced after target enrichment with the 1052-amplicon panel. **D)** The 2x2 table showing maximum performance for samples sequenced after target enrichment with both the AmpliSeq and 1052-amplicon panels. **E)** The 2x2 table showing maximum performance for samples sequenced after target enrichment with the AmpliSeq panel only.

SVs are largely technology-specific artefacts (particularly driven by the library prep and panel) that limit a panel’s sensitivity as variant caller thresholds must be increased to mitigate the false positive rate (FPR). This concept is more critical for novel variant and mutation discovery where filtering out unannotated sites is unacceptable or a normal-tumor comparison is not feasible. We reasoned that variant confirmation using an alternative technology could reduce false positives. As a proof-of-concept, we evaluated the potential for the 1052-amplicon panel to identify variants in the 6 reference mixtures, with and without sequential testing using an alternative sequencing strategy, namely AmpliSeq Cancer Panel (Life Technologies) enrichment followed by sequencing on a PGM. Because the 1052-amplicon panel content is much more expansive than the AmpliSeq panel, we considered only genomic positions common to both platforms. As a baseline, we used simple percent variant to call variants and excluded sites with insufficient sequencing coverage on either platform (<100 reads and <0.1 median depth of coverage). The f-measure, the harmonic mean of sensitivity and PPV, was used as the performance metric. This metric is appropriate when one is interested predominately in the performance of a single class especially those with a low prevalence [42]; in this case, most genomic positions are wild-type and approximately 1000-fold fewer positions are SNPs or mutations (positives). Sequencing the same reference DNA samples on different platforms revealed SVs specific to both platforms relative to expected positives (Figure 4A). Note that the SVs identified previously were included for this analysis. The f-measure was maximized at 0.67 on the 1052-amplicon panel at a 6% threshold, where variant percentages above 6% are predicted positives and variants below 6% are predicted negatives (Figure 4B and C). However, when the percent variant threshold was allowed to vary independently between the 1052-amplicon and AmpliSeq panels such that a positive is predicted if the percent variant exceeds the thresholds for both panels, the f-measure was maximized using a 2% threshold for both panels (Figure 4B and D). This outcome was not the result of superior performance of the AmpliSeq panel compared to the 1052-amplicon panel. Indeed, the f-measure for the AmpliSeq panel was maximized at 0.55 at a 27.5% threshold, a value that is appreciably lower than that of the 1052-amplicon panel (Figure 4E). Instead, it reflects the independence of SV’s inherent to each TAS methodology.

As expected, TAS platform performance was directly affected by excluding the previously derived SVs: the optimal f-measure for the 1052-amplicon panel climbed from 0.67 at 6% to 0.79 (note that the SVs were derived from an independent data set). Including a variant confirmation step in the workflow (here, the the AmpliSeq panel) increased PPV by identifying additional platform-specific false positives that were not previously identified, while simultaneously increasing sensitivity (that is, the detection sensitivity) by allowing variant calling thresholds on 1052-amplicon panel results to be decreased. To be clear, the analytical sensitivity of each platform is still the same and the maximal analytical sensitivity achieved from both platforms will be limited by the least sensitive platform. But realistically, the analytical sensitivity of each platform is increasingly realized because the FPR was mitigated by the orthogonal platform. Although running independent sequencing workflows in a discovery context is not always feasible, these results show that initial variant calling thresholds can be relaxed if variants will be confirmed using an alternate method. This approach greatly increases the PPV of detected variants while exploiting the intrinsic analytical sensitivity of the system.

### Evaluating panel performance with FFPE DNA samples

**Figure 5:**
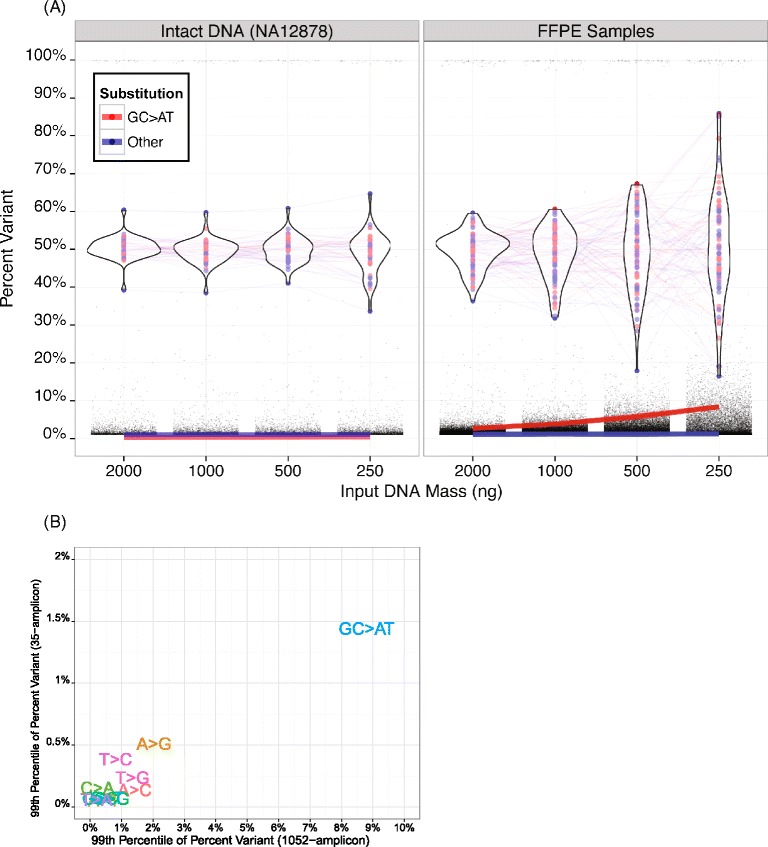
**The effects of input DNA quality and quantity on variant detection background distributions. A)** The figure shows variant calling background for the indicated mass amounts of intact cell line or comprised quality, FFPE sample DNA. The standard deviation of the heterozygous variants increases only slightly with reduced DNA input quantities with intact DNA (left panel), but increases dramatically when the quantity of lower quality, FFPE DNA is reduced (right panel). The 99th percentile of the background percent variant (red and blue lines) is more consistent for cell line DNA than for FFPE DNA with the rise in the background being largely driven by the GC > AT transitions. **B)** The median 99th percentile of the background for all possible substitutions (with G > A and C > T collapsed into GC > AT) from TAS analysis using 250 ng DNA from 8 FFPE samples using the 1052-amplicon panel (x-axis) compared to after target enrichment using an independent 35-amplicon panel (y-axis). As expected, GC > AT transitions contribute higher background than other possible substitutions.

Reference intact DNA mixtures are informative standards for establishing lower limits of detection while also estimating the linearity and accuracy of the panel. However, for most oncology applications, the ability to analyze FFPE samples is crucial. DNA from two FFPE samples and one intact cell line (NA12878) were sequenced to estimate the effects of input DNA on the behavior of heterozygous variants and background distribution (positions that match the reference genome) in the TAS system. Heterozygous variants averaging approximately 50% percent variant were identified from 2000 ng FFPE DNA input and tracked in a titration study with as little as 250 ng DNA input (Figure 5A). Whereas the standard deviation of the heterozygous variants called increased from 3.0 to 5.5 with decreasing quantities of intact cell line DNA (86% increase from 2000 ng to 250 ng input), the effect was far more dramatic for the two FFPE DNA samples, increasing from 5.1 to 15.6 and 4.4 to 12.4 (a 205% and 185% increase from 2000 ng to 250 ng input). Not surprisingly, the absolute magnitude of the standard deviations was also much larger for the FFPE samples. The standard deviations were slightly improved when heterozygous variant calling was limited to positions with ≥500 reads of coverage (data not shown). As expected, the variability of the heterozygous variants was not dependent on whether the substitution is a GC > AT transition; this was in contrast to behavior of the background distributions.

Similarly, the background percent variant increased with decreasing quantities of input DNA. The 99th percentile of the variant background was 2.6% and 2.7% for the two FFPE samples at 2000 ng, but increased to 8.4% for both FFPE samples at 250 ng input. The background percent variant was also consistently elevated between library preps for GC > AT transitions when we compared the 99th percentiles of results from 8 FFPE samples sequenced using either the 1052-amplicon panel or an independent 35-amplicon panel [24] (Figure 5B). When considering only common regions with at least 100 reads and sequencing depth ≥0.1 of the sample median depth of coverage, we observed that the median percent variant of GC > AT transitions at the 99th percentile across 8 FFPE samples was 8.8% for the 1052-amplicon panel and 1.4% for the 35-amplicon panel. Sequencing data from samples enriched using the smaller 35-amplicon panel comprise a lower background percent variant than from the broader panel, but the amplicons were shorter, the sequencing depth was an order of magnitude greater (10,000X–30,000X sequencing depth), and the input requirements were distinct. This result suggests that the background calling of transition mutations can be variable between target enrichment methods and DNA input quantity, but will be consistently higher than other types of substitutions within a given panel, library prep, and platform.

### Optimizing variant calling for FFPE

Since GC > AT transitions have a higher background than other substitutions in FFPE samples, variant calling must account for this noise profile. The simplest strategy is to set independent thresholds for GC > AT and all other hypotheses at the risk of a loss of sensitivity or PPV or both. We implemented a more flexible approach based on sample- and hypothesis-specific cutoffs whereby the thresholds were set to match SNP rediscovery rates stratified by GC > AT status (see Materials and Methods). This strategy was founded on the expectation that most variants are previously identified (and annotated) and predominately germline as opposed to somatic. Indeed, this approach is supported by previous research, which found that mutation rates in lung adenocarcinoma [43] and colorectal cancer [44], two of the most mutated cancer genomes, are on the order of 1–10 somatic mutations per megabase compared to ~1 event per kilobase for germline variants [45]. We imposed an additional constraint based on our observations that higher background can be expected for GC > AT transitions than for other base substitutions. First, we tested our variant calling strategy on the sequencing data from the FFPE DNA titration described above (Table 3). As expected, the thresholds for predicting a variant increased as the input DNA amount decreased, with a concomitant increase in background, particularly for GC > AT transitions. A subset of the variant calls was intersected with confirmed variants from a 35-amplicon panel to estimate performance in an approximate 3 kb region of overlap. The sensitivity and PPV remained at 100% until the input was reduced to 250 ng, at which point 3 false positives were called between the two FFPE samples (2 of the 3 false positives were C > T transitions). At that point, PPV was reduced to 80% and 71% for the two FFPE samples while maintaining 100% sensitivity.

**Table 3:** **Concordance between the 1052-amplicon and 35-amplicon panels as a function of input DNA mass and adaptive thresholds**

**FFPE sample**	**Input DNA mass (ng)**	**Platform concordance**			**Variant calling with the 1052-Amplicon panel**				
					**Variant caller threshold**		**Percent annotated**	**Ti/Tv ratio**	**Variants/kb**
		**FN**	**FP**	**TP**	**GC > AT**	**Other**			
1	2000	0 (0)	0 (0)	5 (5)	6 (6)	6 (6)	88% (88%)	2.72 (2.72)	0.89 (0.89)
	1000	0 (0)	0 (0)	5 (5)	7 (6)	6 (6)	79% (63%)	3.48 (4.74)	0.98 (1.26)
	500	0 (0)	0 (4)	5 (5)	8.5 (6)	7 (6)	80% (18%)	2.35 (12.72)	0.93 (5.10)
	250	0 (0)	2 (10)	5 (5)	8.5 (6)	7.5 (6)	82% (11%)	2.18 (17.34)	0.85 (11.19)
2	2000	0 (0)	0 (0)	4 (4)	6 (6)	6 (6)	80% (80%)	2.83 (2.83)	0.88 (0.88)
	1000	0 (0)	0 (2)	4 (4)	7.5 (6)	6 (6)	84% (56%)	2.67 (4.79)	0.84 (1.32)
	500	0 (0)	0 (8)	4 (4)	8 (6)	7 (6)	83% (16%)	2.96 (15.03)	0.83 (5.47)
	250	0 (0)	1 (9)	4 (4)	8 (6)	8 (6)	77% (10%)	3.40 (17.43)	0.84 (10.17)

The indicated mass amounts of two FFPE samples were sequenced after target enrichment with the 1052-amplicon panel. For comparison, 2000 ng DNA from each sample was also sequenced using an alternative 35-amplicon TAS panel. The true variants were defined based on sequencing results from the 35-amplicon panel: false negatives (FN) are variants missed by the 1052-amplicon panel; false positives (FP) are variants not detected by the 35-amplicon panel; and true positives (TP) are variants called by both panels. In general, the variant calling thresholds adaptively increase to adjust to the higher backgrounds of variants detected with the lower input DNA mass amounts. The thresholds are set independently for GC > AT hypotheses versus all other hypotheses; they are based on the log of the variant caller score. The adaptive threshold strategy satisfies multiple criteria spanning all positions of the 1052-amplicon panel with respect to: maintaining a high percent of annotated variants (a surrogate for PPV when the true genotype is unknown); acceptable transition to transversion ratio; and acceptable number of variants called per kb. The parenthetical numbers are the results from maintaining a constant (non-adaptive) threshold. Note that the non-adaptive thresholds remain constant to show the drop in percent annotated and the increased call rate. If the thresholds were held constant at, say, 8, then the call rate would decrease suggesting a drop in sensitivity (data not shown).

A surrogate estimate for PPV is the percent of the called variants annotated by either dbSNP or COSMIC; these estimates remained stable or dropped slightly at the lowest amount of input DNA. The results suggested that the thresholds climbed as expected to mitigate false positives (particularly for GC > AT transitions), thus balancing high sensitivity and PPV. If variant caller thresholding did not adapt to the increasing background from low input or low quality samples, then the PPV would drop significantly due to the increase in the false positive rate (Table 3). The opposite is also true. That is, if the thresholds for low quality samples were applied to high quality samples, the sensitivity and false positive rate would drop.

A previous study showed the false positive rate in variant calls was not found to be higher from FFPE compared to frozen samples [37], but those results were generated using a genotype-calling application rather than low-level variant detection. Interestingly, the study also found elevated GC > AT transitions compared to other substitutions. In our observations, the high background became a dominating factor for lower-level variant detection based on the behavior of the tail (such as the 99th percentile) of the background distribution. With relatively low-quality DNA samples and low amounts of available DNA, it was important to maximize sequencing depth to mitigate false positives (as suggested by Kerick et al. [12] for sequencing studies <100X coverage), although the pool of amplifiable DNA is finite in such samples.

### Using the QFI as a pre-analytical tool for risk mitigation and sample prioritization

Too often, there is a negative correlation between the clinical relevance of samples and their suitability for NGS. In many cases, precious samples tied to critical clinical endpoints are low in DNA abundance and/or quality, and are not standardized with regard to collection and nucleic acid isolation methods. For this reason, we recently developed a preanalytical sample qualification assay (QFI) that quantifies the absolute number of templates available for amplification in a DNA sample and predicts sample performance in downstream NGS assays [21].

**Figure 6:**
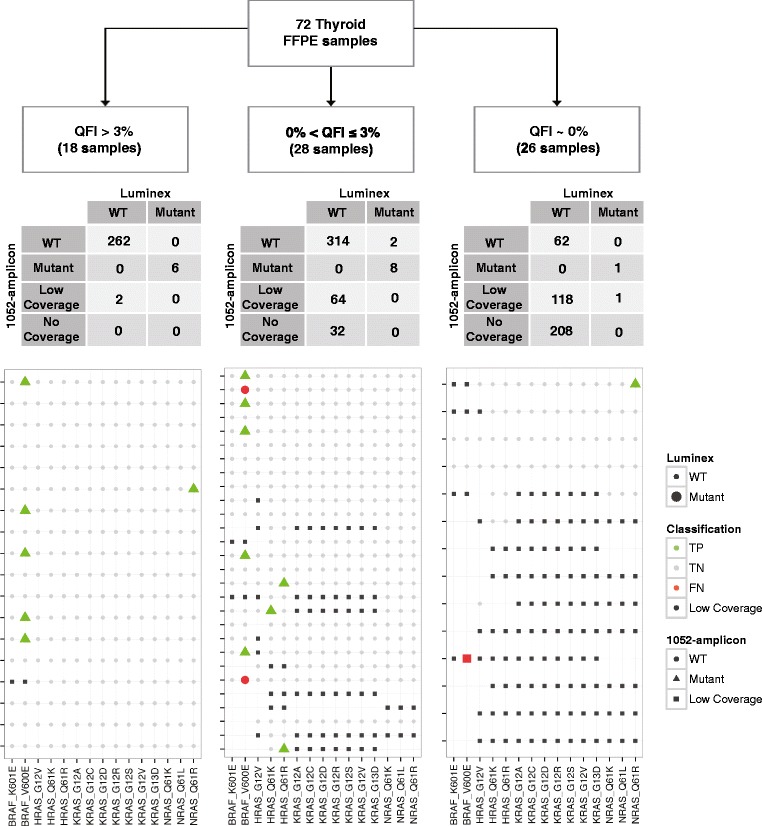
**Interplatform variant dection concordance stratified by QFI.** DNA from 72 thyroid cancer FFPE samples was used to test the concordance of variant calling results between 1052-amplicon panel and a liquid bead array system (Luminex). The samples with the highest quality DNA (QFI >3%) showed the strongest concordance, >99% between the two variant detection platforms. The concordance rate decreases with reduced QFI. The size of the markers is based on Luminex status (large markers are mutants, small markers are wild type). The color of the markers is based on the classification status of the 1052-amplicon panel with data from the Luminex platform defined as truth: green is for true positives (TP); gray is for true negatives (TN); orange is for false positives (FP); red is for false negatives (FN); and black is for low sequencing coverage by the 1052-amplicon panel (Low Coverage). The specific predictions from the 1052-amplicon panel are defined as wild type (circles), mutant (triangle) and low coverage (square). The number of rows in the agreement maps corresponds to the number of samples in the study except when a sample had no coverage at any of the 15 specified hypotheses. For example, the left most panel has 18 rows corresponding to all 18 samples that were sequenced (with a total of 15 hypotheses *18 samples = 270 entries) so the 4x2 table above the agreement map sums to 270. However, the right most panel only has 14 rows representing 26 samples. That is, 12 samples had no coverage associated with the 15 hypotheses and are not shown, but the corresponding 4x2 table still sums to 15 hypotheses *26 samples = 390 entries. Incidentally, none of the samples that had zero coverage by the 1052-amplicon panel workflow had positive calls using the Luminex method.

To further assess the value of the QFI within the 1052-amplicon NGS workflow, quantify the risk in sequencing low quality samples, and challenge the analytical performance of the 1052-amplicon panel, we evaluated a challenging set of 72 clinical thyroid FFPE biopsies with block ages ranging from 1 to 19 years (median age = 15 years). Of the 72 samples, only 18 (25%) met the passing QFI threshold of >3% amplifiable templates. The remaining samples either had no detectable QFI (n = 26) or a QFI that was measurable below 3% (n = 28). These samples were sequenced using the 1052-amplicon panel and results were compared to those obtained using a liquid bead-based assay (Signature® KRAS; Asuragen, Inc.) with a validated analytical sensitivity of 1% [46] (see Figure 6). The comparison focused on call concordance at 15 sites across the *BRAF*, *HRAS*, *KRAS* and *NRAS* genes, corresponding to the most common mutations in thyroid cancer.

As expected, QFI scores were associated with sequencing uniformity and depth using the 1052-amplicon panel (Additional file 1: Figure S4). Genetic variants detected in the 18 samples with a QFI score >3% demonstrated strong concordance with those seen with the Signature® mutation assays; both methods correctly identified 6/6 (100%) variants. Five of the mutations were associated with *BRAF*, the most prevalent mutation in thyroid cancer, and 1 mutation was in *NRAS*. Sequencing depth for one sample with a QFI of 3.9%, however, was lower than expected (<100 reads) for *BRAF*. For the remaining 28 samples with measurable QFI estimates, concordance between the platforms was apparent but diminishing. Variant detection sensitivity of the 1052-amplicon panel was 78%, with 7/9 variants identified, but 64/420 (15%) and 32/420 (5%) of the hypotheses of interest had low (<100 reads) and no coverage, respectively. The two false negatives were associated with *BRAF*; percent variant estimates from TAS were near baseline for both variants. Importantly, the two variants associated with *HRAS* would not have been identified in this study if the samples with a measurable QFI less than 3% had been excluded. These samples represented a gray area in terms of suitability for analysis; relevant mutations could be confidently identified, but there was a significant risk of coverage loss (20% (96/420)) of the genomic positions of interest had low or no coverage) and inaccurate variant calling (2 false negatives and 1 false positive). The low QFI could be treated as an indicator that DNA input should be increased–a strategy that has been shown to rescue low-quality DNA samples [21]. Despite the variant call inaccuracies, 5 of 7 detected variants in samples with QFI <3% were in the *BRAF* gene, suggesting that sequencing coverage loss would not preclude the sample utility in a biomarker discovery context.

The last stratum of this sample cohort was associated with an undetectable QFI, indicating that less than 1 amplifiable copy per 150 input templates was present [21]. The sensitivity of mutation detection from this group could not be accurately assessed since only two mutations were known (one was reported by TAS). Many of the variant genomic positions had low (118/390 = 30%) or no (208/390 = 53%) coverage.

Since QFI measures the proportion of DNA template accessible for amplification, we investigated whether the detection rate of genetic variants was a function of QFI. Of the 72 thyroid samples for which we obtained both TAS and Signature® assay data, 18 were classical papillary thyroid carcinomas (PTCs), which have a high prevalence of *BRAF* mutants [47]. We observed an insignificant association when stratifying *BRAF* status as determined by TAS (mutant versus wildtype) by QFI (p-value = 0.09 by Fisher’s test). However, if we expanded the sample set to all 30 PTCs (Table 4), *BRAF* detection rates from both TAS and the Signature® assay were significantly associated with QFI (p-value = 0.005 and p-value = 0.03 by Fisher’s test, respectively). That is, platform concordance was strong, but both platforms detected fewer variants in the lowest-quality (QFI) samples. The implication is that any assay for variant detection based on PCR will likely suffer a drop in sensitivity with low-quality samples [21]. Furthermore, this should be carefully considered when evaluating platform agreement (consistency) and variant detection (accuracy).

**Table 4:** **Association between** ***BRAF*** **detection rate and QFI status for PTC samples**

**All PTC samples, N = 30**				
	**Luminex**		**1052-amplicon**	
	**BRAF WT**	**BRAF mutant**	**BRAF WT**	**BRAF mutant**
QFI = 0%	9	1	10	0
0% < QFI < = 3%	6	7	8	5
QFI > 3%	2	5	2	5
Fisher-test p-value	0.03		0.005	

When considering the 30 PTC sample subset of the 72 sample Thyroid set, both the Luminex and the 1052-amplicon panel showed a significant association between BRAF detection and QFI status.

The association between high background and reduced input of PCR-competent DNA can, to some extent, be explained by QFI. As the QFI decreased, the variant background increased and greater variability in the detection of heterozygous variants was observed (Additional file 1: Figure S5); this result is conceptually similar to the findings from the FFPE-DNA titration study (Figure 5). However, only homozygous variants could be reliably detected for samples with undetectable QFI, because the underlying template diversity was so low that the probability of sampling the true template distribution significantly decreased. This created an artificially low background distribution, poor quantitation, and loss of sensitivity for all variants except homozygous variants.

We speculate that previous studies that observed consistent results from FFPE and fresh frozen samples [13] may have involved FFPE samples with relatively high functional quality, which, as a result, behave more like intact DNA samples. Clearly, the QFI can provide specific guidance for sample inclusion and exclusion for clinical studies. Organizations such as Genome in a Bottle (http://www.genomeinabottle.org/) and the International Cancer Genome Consortium (http://www.icgc.org/) provide specific recommendations about controls, standards, and statistical analysis, but currently do not address FFPE samples. The implications from our work suggest using QFI for sample qualification. We have also shown that variant detection can be quantitatively affected by FFPE sample quality. As a result, assumptions about mutational prevalence and any subsequent power analysis for detection must be qualified. Ultimately, the QFI offers quantitative insights into the trade-offs between sensitivity and PPV and can be integrated into a comprehensive NGS workflow to optimize call performance and provide increased flexibility to address the specific goals of the TAS application, such as clinical research or patient testing.

### Sequencing clinical FFPE colorectal samples

Evaluation of a discrete cohort of colorectal cancer (CRC) specimens was undertaken to provide a more holistic perspective of the challenges we addressed one-by-one during development of the 1052-amplicon TAS panel. To that end, we procured 26 FFPE CRC samples to create 24 DNA mixtures and 22 neat samples (see Materials and Methods). Of the 46 FFPE samples, 26 had *KRAS* codon 12/13 mutations to identify to characterize performance of the entire workflow: sample qualification, target enrichment, sequencing, variant calling and variant confirmation. Note that all 46 samples were sequenced using the 1052-amplicon panel. The performance of the targeted NGS workflow was evaluated in terms of the detection sensitivity and PPV of the expected *KRAS* variants. DNA samples (250 ng) were evaluated to determine suitability for target enrichment and sequencing using QFI. As per previous guidelines [21], samples with a QFI <3% were rejected from analysis, leaving 43 samples (43/46 = 93% pass rate). Of these, 2 samples did not yield reliable variant calling thresholds, i.e., their thresholds could not be set in a sample-specific manner in order to satisfy the constraints for percent annotated, transition/transversion (Ti/Tv) ratio, and calls per kilobase (41/43 = 95% passed threshold analysis) (see Materials and Methods). In the end, 41 out of 46 samples (89%) passed both QFI and analysis qualifications: 18 of the 22 neat samples and 23 out of the 24 mixtures.

**Figure 7:**
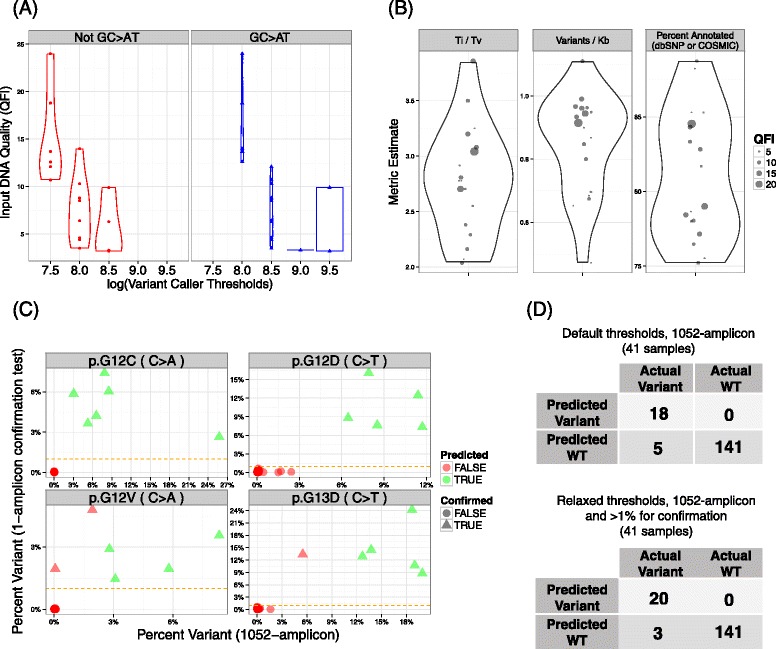
**Variant calling summary for FFPE CRC samples. A)** Violin plots of the variant calling thresholds (x-axis) for each sample stratified by hypothesis group: GC > AT transitions (blue right panel) versus all other substitutions (left red panel). The thresholds are higher for samples with reduced QFI estimates (y-axis) and for GC > AT hypotheses. The width in the plots correspond to the density of points in the region. **B)** Violin plots of metrics associated with SNP rediscovery. The transition to transversion (Ti/Tv) ratios, variants called per kilobase, and the percentage of variants annotated by dbSNP or COSMIC are shown in the panels from left to right. All metrics are independent of QFI based on univariate analysis, except the number of variants called per kilobase which is significantly negatively correlated with QFI. **C)** Plots of the percent variant for each of the *KRAS* alleles as measured by the single amplicon confirmation platform (y-axis) as a function of the percent variant found using TAS with the 1052-amplicon panel (x-axis). In this plot, the predicted and confirmed variants were called using relaxed thresholds for the 1052-amplicon panel method results with confirmation at 1% by the single-amplicon assay sequenced on the PGM (horizontal orange dashed line). **D)** 2x2 tables showing classification performance of *KRAS* variant prediction from TAS with the 1052-amplicon panel stratified by default threshold analysis (upper 2x2 table) or incorporation of confirmation testing and relaxed thresholds (lower 2x2 table). There are 41 samples considered for analysis spread across 4 *KRAS* alleles giving 4*41 = 164 total hypotheses tested.

The variant caller thresholds for the 18 neat samples are shown per sample, stratified by hypothesis group, in Figure 7A. To maintain consistency with SNP rediscovery rates, the variant caller thresholds for GC > AT were higher than those for the other substitutions, as were the thresholds for the noisy samples presenting lower QFIs. This strategy mitigated the risk of a high false-positive call rate from low-quality samples, as seen in previous studies [21]. A significantly positive association (p-value = 0.03 by SRC) was observed between the number of variants called per kilobase and the QFI score for the 18 neat samples (Figure 7B). The call frequency can be explained by examination of the other two metrics shown in Figure 7B. In order to keep these two metrics, Ti/Tv ratio and percent annotated variants in dbSNP or COSMIC, acceptably constrained for lower quality, lower QFI samples, the number of variant calls must decrease; otherwise the percentage of annotated variants (a surrogate for PPV) would plunge. This analysis was driven by the demand to maintain reasonable SNP rediscovery rates.

Using all 41 qualified FFPE CRC samples, we next examined the implications of this variant calling strategy by evaluating the sensitivity and PPV of variant detection in two clinically actionable *KRAS* codons (even though the thresholds for variant calling set for the entire 109 kb panel are not locus-specific) (Figure 7C and D). The known variant frequencies ranged from 0.1% to 25.8% (median = 7.9%) as measured using the 1052-amplicon panel, with sensitivity and PPV of 18/23 = 78% (95% CI = 56.3, 92.5) and 18/18 = 100% (95% CI = 81.5, 100.0), respectively. The 5 false negatives (FNs) in this experiment had variant frequencies of 0.1% to 5.5% (median = 2.8%). Of the 18 predicted variants, 16 were confirmed with the previously established 2% threshold (based on the analysis in the previous section) in an alternate confirmation test using singleplex target enrichment and sequencing on the PGM. Hence, the net sensitivity was 16/23 = 69.5% (95 CI = 47.1%, 86.8%), while maintaining 16/16 = 100% PPV (95 CI = 79.4%, 100.0%). However, the thresholds for the 1052-amplicon panel were derived without anticipation of variant confirmation using an independent method. Our previous results strongly suggest that improved performance can be achieved if confirmation testing is integrated within a comprehensive analysis strategy. Thus, variant calling thresholds could be relaxed, here by 20%, and the confirmation threshold could be decreased to 1%, in which case sensitivity would increase to 20/23 = 87.0% (95% CI = 66.4,97.2) with 20/20 = 100% PPV (95% CI = 83.2,100.0). The consequence of relaxed constraints was that the median percent variants annotated of the 41 samples decreased from 79% (IQR = 8.2%) to 68% (IQR = 8.6%) with a concomitant rise in the number of variants called per kb. These analyses demonstrate the balance and trade-off for sensitivity and PPV with and without the context of threshold relaxation coupled with confirmation testing.

## Conclusions

In this study, we used intact cell line DNA samples, and low- and high-quality FFPE samples in contexts ranging from controlled analytical studies to clinically relevant and well-characterized oncology sample cohorts to evaluate the performance of a TAS system that encompassed sample qualification and quantitative QC, a 1052-amplicon pan-cancer enrichment panel, and a comprehensive bioinformatics pipeline and reporting strategy. Our specific approach utilized defined analytical methods (i.e., custom cell mixtures to address accuracy, linearity and precision), multi-tier bioinformatics analyses and tools (i.e., systematic variant identification, variant caller performance and an HTML interface for visualizing and navigating results), distinct sequencing strategies (orthogonal platform comparison, sequential testing effects on accuracy) and clinically relevant evaluations and applications (sequencing, analysis and interpretation of two large independent FFPE cohorts). With a DNA input of 250 ng, the linearity was 0.99 based on the CCC and 4 independent sequencing runs showed an interquartile range of 1.52% for variants at 2%. By incorporating results from an orthogonal sequencing platform, we not only confirmed predicted SVs (Wilcox signed-rank test p-value <10^−16^), but we also demonstrated the increase the f-measure of TAS up to 51%. We reiterated the utility of the QFI with two indepenedent FFPE cohorts to show that coverage and inter-platform concordance are positively correlated with the pre-sequencing QFI metric. We extrapolated the concept of increased performance through confirmation testing from cell lines to FFPE to increase sensitivity from 78% to 87% while maintaining 100% PPV. The results underscore the value of an integrated clinical workflow for targeted NGS.

More recently, increased focus has turned to simultaneous identification of multiple types of aberrations such as copy number variation (CNV) in addition to indel and SNV detection [15]. Importantly, the concepts for SNV detection presented here can also be extended to indel detection with the caveat that more mixtures and samples will be necessary to achieve a comparable number of aberrations. CNV analysis has had more visibility using hybrid-capture based workflows compared to PCR based workflows [15]. We are currently improving the bioinformatic analysis and experimental workflows to better represent the range of aberrations and analytes available for measurement.

Finally, we note that the increasing commoditization of WGS and WES provides alternatives to TAS for clinical resequencing applications. In particular, WGS offers the most comprehensive content, established and straightforward workflows, and well-characterized pipelines and tools for use with high-quality DNA to capture the full range of sequence variation, including copy number changes, structural rearrangements, and indels. With our limited understanding of the clinical interpretation of genome-wide variation in oncology, however, the strengths of WGS can also be a liability. WGS reveals millions of variants in each patient sample, including large numbers of alterations that may require tedious review to determine their clinical significance, if any. For this reason, WGS of tumor-normal pairs is often advisable, but this approach further escalates the costs compared to TAS and is still may be confounded by “driver” vs. “passenger” mutations. Even as the costs of WGS decline, TAS still offers a number of advantages, including sample throughput and sequencing depth to detect low-abundance, clinically actionable variants in challenging specimens such as FFPE. To this point, the number of cancer-related variants with compelling evidence to guide patient management (and reimbursement) based on available therapies and interventions is actually quite modest, and most clinical laboratories favor panels that target 20–50 genes. As a result, a Illumina MiSeq or an Ion Torrent PGM or Proton sequencer are appropriately scaled to their needs. A number of other advantages for TAS compared to WGS or WES for clinical diagnostics have been detailed elsewhere [48]. Last, we note that orthogonal sequencing to improve call accuracy—an approach that we highlight here using TAS—has also been reported for WGS [49]. Yet, again, many of the benefits of TAS are expected to persist compared to WGS due to the ~3-6 orders of magnitude reduction in content (while still retaining the most clinically relevant sequences) and associated reduction in bioinformatic and interpretative complexity. Fundamentally, TAS is better suited to report well-characterized mutations that are known to be actionable as a first-line test, without the limitations of low-coverage WGS that can overlook these mutations in heterogenous tumor specimens. In situations where TAS fails to report clinically meaningful molecular information, WES or WGS may be viable options by “casting a broader net” in some cases.

In summary, our results highlight the value of a comprehensive diagnostic approach to target amplicon sequencing that integrates preanalytical, analytical, and postanalytical quality measures and analyses, and offers reliable detection of clinically relevant mutations from challenging tumor specimens. We suggest that the emergence of TAS as the method of choice for the “first wave” of cancer diagnostic NGS assays requires similar strategies for process integration to combat erroneous interpretations, expand options for the accurate assessment of low-quality tumor biopsies, and ensure reliability in routine patient testing and in individualizing therapy choices.

## Additional file

Additional file 1:
**Figures S1–S7.** This is a Microsoft word document containing images and legends that describe selected findings from the research that were deemed less important to the overall communication than the figures included in the main body of the manuscript.

## Abbreviations

NGS: Next-generation sequencing
TAS: Targeted amplicon sequencing
SV: Systematic variant
PPV: Positive predictive value
Ti: Transition
Tv: Transversion
WGS: Whole genome sequencing
WES: Whole exome sequencing
PGM: Personal Genome Machine
FFPE: Formalin-fixed paraffin-embedded
SRC: Spearman rank correlation
CCC: Concordance correlation coefficient
FPR: False positive rate
PTC: Papillary thyroid carcinoma
CRC: Colorectal cancer
AP: Average precision
FN: False negative
FP: False positive
TP: True positive, hemotoxylin and eosin (H&E)
