# Supplementary material for: Evaluation of an integrated clinical workflow for targeted next-generation sequencing of low-quality tumor DNA using a 51-gene enrichment panel
Source: BMC Med Genomics. 2014 Nov 14;7:62. doi: 10.1186/s12920-014-0062-0 (PMC4241214; doi:10.1186/s12920-014-0062-0)
Supplement: Additional file 1: — Figures S1–S7. This is a Microsoft word document containing images and legends that describe selected findings from the research that were deemed less important to the overall communication than the figures included in the main body of the manuscript. [file 12920_2014_62_MOESM1_ESM.docx]

# Supplemental Information for “Evaluation of an integrated clinical workflow for targeted next-generation sequencing of low-quality tumor DNA using a 51-gene enrichment panel”


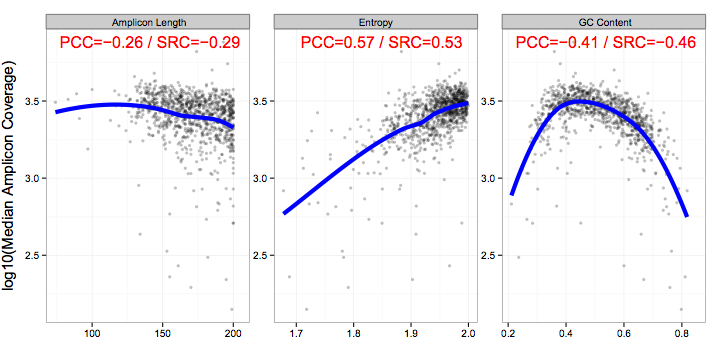


**Figure S1. Association Between Sequencing Depth and Sequence Characteristics**

Univariate associations between different sequence characteristics, amplicon length, sequence entropy, and GC content (x-axis); and the log10 of median depth of coverage (y-axis) are shown as black dots. Entropy is a measure from information theory that captures the amount of uncertainty in a sequence of characters (in this case: A, T, G and C). The GC Content is simply the percentage of bases that are either G or C, irrespective of order. The estimates of entropy and GC content are measured at the amplicon level. Univariate estimates of Pearson correlation coefficients (PCC) and Spearman rank correlation (SRC) are shown for each panel and blue lines represent loess-smoothed estimates.

**Figure S2. Linearity and Accuracy as a Function of DNA Input**

Plots of the observed (y-axis) versus expected percent variant (x-axis) for all annotated genetic variants in the reference cell-line DNA mixtures, stratified by input DNA mass. The size of the markers is associated with the sequencing depth observed, and the color is associated with the specific mixture. Note that all
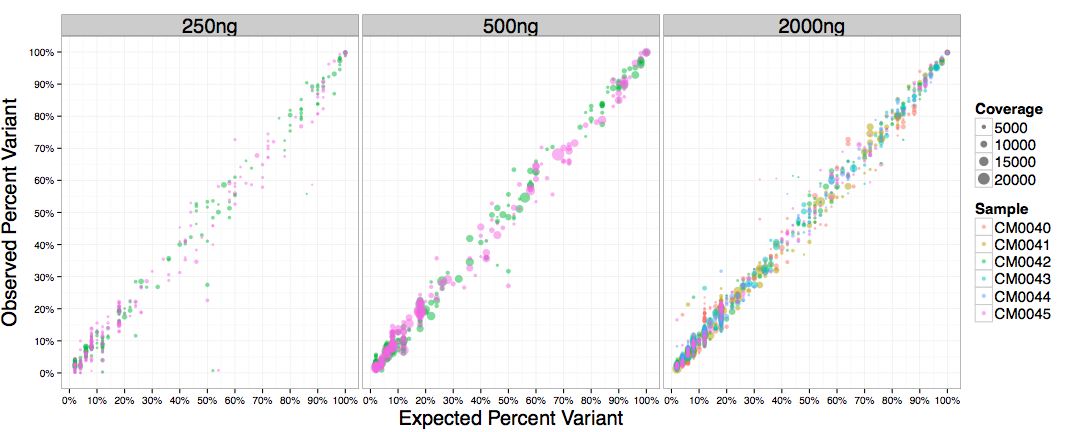
 6 mixtures were sequenced at full 2000 ng DNA input while two mixtures were also sequenced at the lower 250 ng and 500 ng inputs. In addition, the coverage was lower for the 250 ng input, hence the marker sizes are smaller and there is more sporadic variant drop out. Only positions with at least 100 reads of coverage and at least 0.1 times the sample median depth of coverage are shown.

**Figure S3. Implications of Filtering on Variant Caller Performance**

A) The average precision of multiple variant callers stratified by sequencing depth, systematic variant (SV) status and annotation. Average precision (AP) (y-axis) is the area under a precision-recall curve where precision is positive predictive value and recall is sensitivity. The x-axis shows the filtering used, including no filtering (None), filtering out positions that are predicted SVs (SV), filtering out data from positions sequenced with less than 0.1 times the sample median depth of coverage (Depth) and filtering using both depth and SV status (Depth + SV). Each point in the plot represents an AP estimate from one of the six reference cell-line DNA mixtures and the solid lines represent the median AP estimate. The left panel is the AP results considering all positions covered by the 1052-amplicon panel, while the right panel on
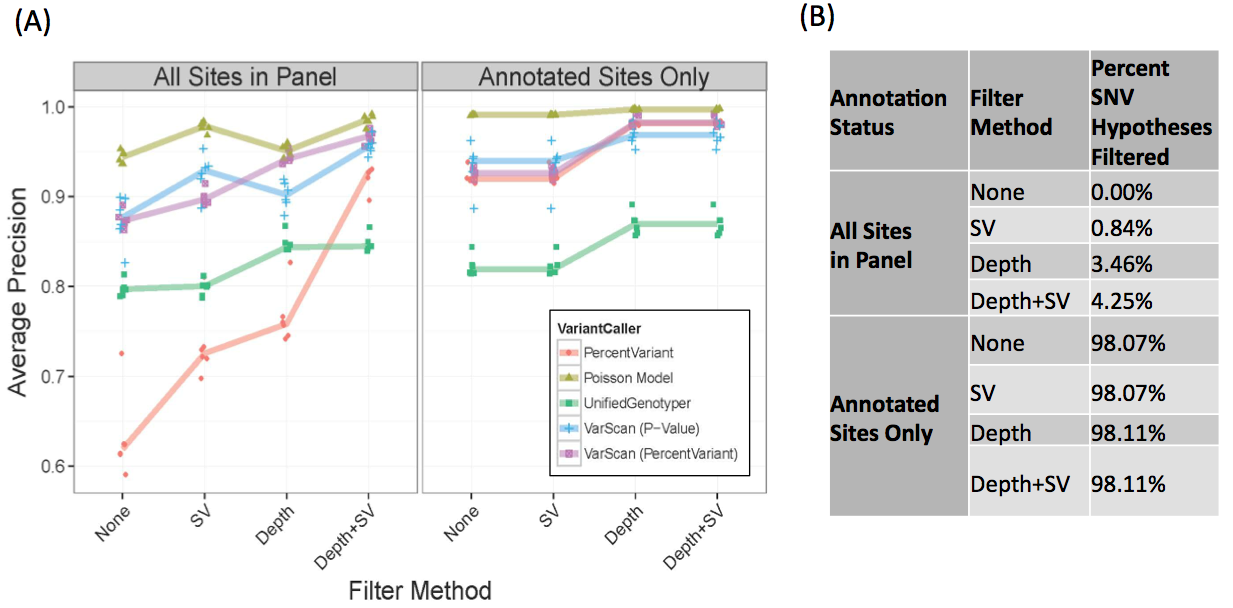
ly considers sites annotated according to dbSNP and COSMIC. In general, we observe that: all variant callers are more precise when both filtering methods are used; raw percent variant provides inadequate precision when used as the sole criterion for variant calling; annotation status can improve variant-calling performance; and some type of filtering is better than none. UnifiedGenotyper is part of the freely available GATK package (v1.3-21) [1, 2] and VarScan (v2.3) [3] is also freely available. The percent variant as reported by VarScan calculates a fraction of variant bases considering only high quality bases using a q-score threshold. The Poisson Model works as previously described [4].

B) The tabulation of hypotheses filtered based on the same methods described in part (A). The percent single-nucleotide variants (SNV) hypotheses filtered is based on the panel interrogating 109,302 genomic positions producing 3*109,302=327,906 testable SNV hypotheses.

**Figure S4. QFI is Associated with Sequencing Depth and Uniformity.**

The association between median sequencing depth (x-axis), uniformity of sequencing depth (y-axis) and QFI (marker shape and color) is plotte
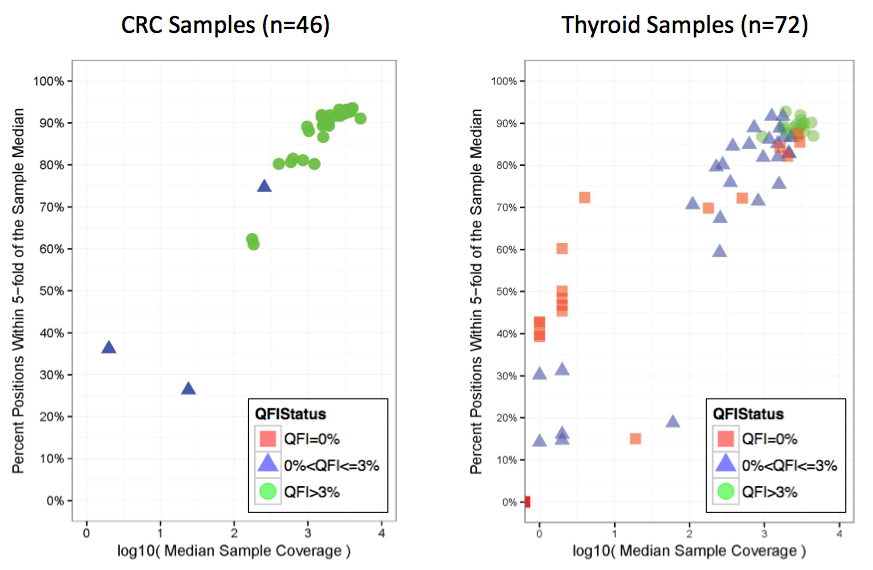
d here. In general, samples from two different cohorts (left and right panels) show a similar trend of association: samples with lower QFI estimates tend to yield lower and more heterogeneous sequencing coverage.


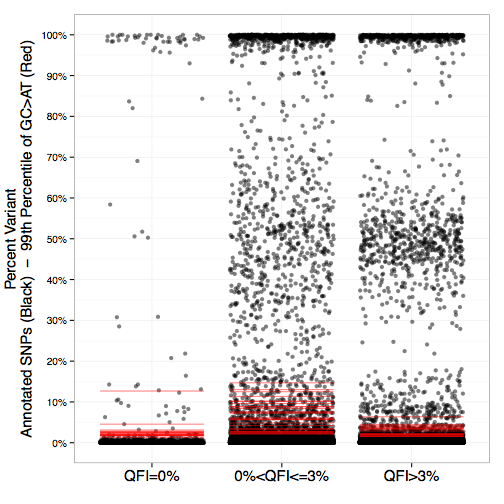


**Figure S5. SNP Variability and GC>AT Background are Inversely Related to QFI**

The figure shows the percent variant (y-axis) of all annotated SNPs according to dbSNP (v132) (black points) and the 99th percentile per sample from all GC>AT transitions (red lines) stratified by QFI (x-axis). For this figure, all samples are pooled together based on QFI (each red line corresponds to a single sample and all black points represent a SNP from a single sample), and only positions with at least 100 reads and >0.1 times the sample median depth of coverage are shown. As a result of the constraints on coverage, the representation from the QFI=0% samples collapses because the sampling of templates for either homozygous-for-reference or homozygous-variants are represented. The probability of the 99th percentile of the background being lower from a sample with a QFI>3% compared to a sample with 0%<QFI<=3% is 18% (Wilcox-test p-value < 0.001)


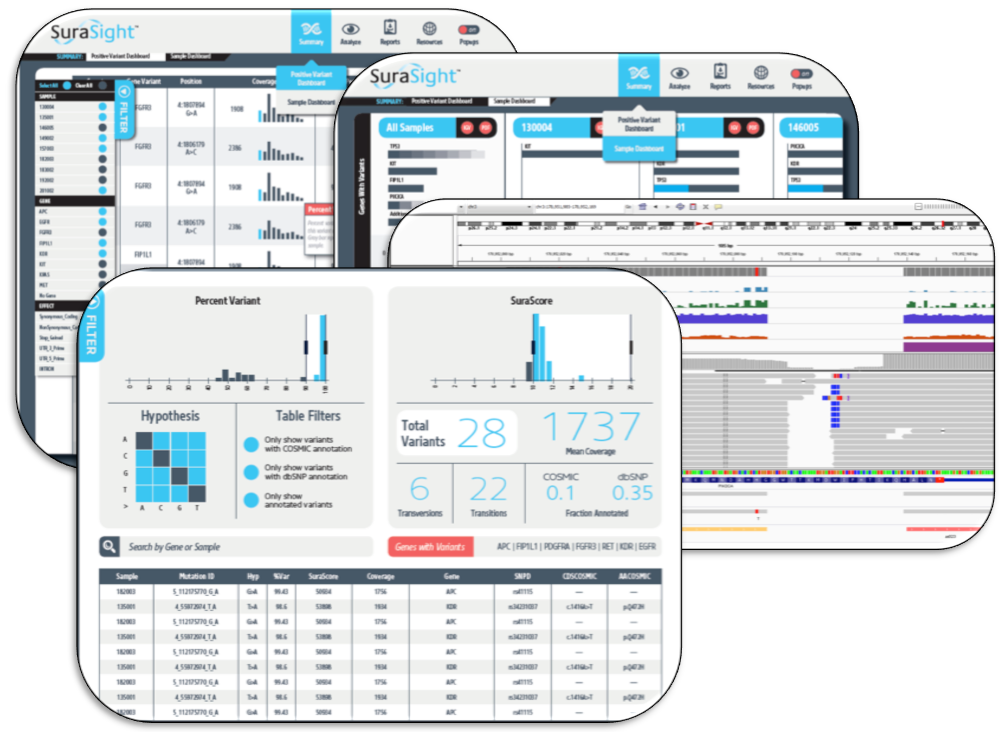


**Figure S6. HTML Interface for Result Navigation and Visualization**

The figure shows snapshots of the Asuragen SuraSight® HTML interface for visualization of TAS results. The interface enables manual inspection of results via PDF report inspection and Integrative Genomics Viewer browser (Broad Institute) navigation.


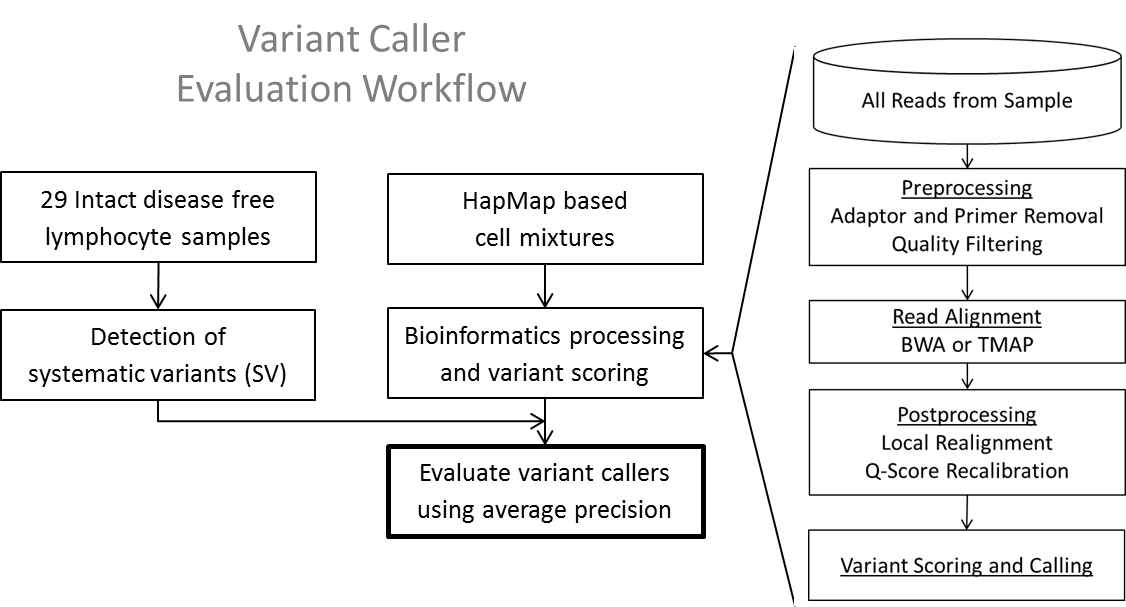


**Figure S7. Analysis Overview for Variant Detection**

The figure shows an overview of the Bioinformatics processing for variant detection. The preprocessing, read alignment and post processing are all consistent with GATK Best Practices circa version 1.3x. The figure also shows how the incorporation of the SV based on an independent set of 29 intact samples and the HapMap mixtures for performance estimates.

**References**

1. DePristo MA, Banks E, Poplin R, Garimella KV, Maguire JR, Hartl C, Philippakis AA, del Angel G, Rivas MA, Hanna M, et al: **A framework for variation discovery and genotyping using next-generation DNA sequencing data.** *Nat Genet* 2011, **43:**491-498.

2. McKenna A, Hanna M, Banks E, Sivachenko A, Cibulskis K, Kernytsky A, Garimella K, Altshuler D, Gabriel S, Daly M, DePristo MA: **The Genome Analysis Toolkit: a MapReduce framework for analyzing next-generation DNA sequencing data.** *Genome Res* 2010, **20:**1297-1303.

3. Koboldt DC, Zhang Q, Larson DE, Shen D, McLellan MD, Lin L, Miller CA, Mardis ER, Ding L, Wilson RK: **VarScan 2: somatic mutation and copy number alteration discovery in cancer by exome sequencing.** *Genome Res* 2012, **22:**568-576.

4. Hadd AG, Houghton J, Choudhary A, Sah S, Chen L, Marko AC, Sanford T, Buddavarapu K, Krosting J, Garmire L, et al: **Targeted, high-depth, next-generation sequencing of cancer genes in formalin-fixed, paraffin-embedded and fine-needle aspiration tumor specimens.** *J Mol Diagn* 2013, **15:**234-247.
